# Status does not predict stress among Hadza hunter-gatherer men

**DOI:** 10.1038/s41598-023-28119-9

**Published:** 2023-01-24

**Authors:** Piotr Fedurek, Julia Lehmann, Laurent Lacroix, Athena Aktipis, Lee Cronk, E. Jerryson Makambi, Ibrahim Mabulla, J. Colette Berbesque

**Affiliations:** 1 School of Human and Life Sciences, University of Roehampton, London, UK; 2 Department of Anthropology, Ludwik Hirszfeld Institute of Immunology and Experimental Therapy, Polish Academy of Sciences, Wrocław, Poland; 3 Health Sciences Research Centre, Roehampton University, London, UK; 4 Department of Psychology, Arizona State University, Tempe, AZ USA; 5 Department of Anthropology, Rutgers University, New Brunswick, NJ USA; 6 Mount Meru Tour Guide and International Language School, Arusha, Tanzania; 7 National Museums of Tanzania, Dar Es Salaam, Tanzania

**Keywords:** Biological anthropology, Behavioural ecology

## Abstract

In recent years there has been much research regarding the extent to which social status is related to long-term indices of health. The majority of studies looking at the interplay between social status and health have been conducted in industrialized societies. However, it has been argued that most of human evolution took place in small, mobile and egalitarian hunter-gatherer groups where individuals exhibited very little variation in terms of material wealth or possessions. In this study, we looked at the extent to which two domains of social status, hunting reputation (being perceived as a good hunter) and popularity (being perceived as a friend), are related to physiological stress levels among Hadza men, hunter-gatherers living in Northern Tanzania. The results of our study show that neither hunting reputation nor popularity is associated with stress levels. Overall, our data suggest that, in at least some traditional small-scale societies exhibiting an egalitarian social model, such as the Hadza, the variation in social status measures based on both popularity and hunting reputation does not translate into one of the commonly used indices of wellbeing.

## Introduction

Social status has been found to be a strong determinant of health in many group-living animals, including human and non-human primates^1^. For example, in Rhesus macaques (*Macaca mulata*), low social status is associated with less effective immune response to infection^2^, while in female yellow baboons (*Papio cynocephalus*) higher dominance status is related to lower glucocorticoids levels^3^. However, studies on non-human primates and other mammals show that the relationship between social status and stress is not straightforward and depends on group composition and within-group sex ratio as well as demography^4–6^. For example, in primate species exhibiting a despotic social model (i.e., with a clear linear dominance hierarchy) dominant males have been shown to have reduced stress levels compared to lower ranking individuals due to better access to resources (and social partners)^7^; however, in some species, such as common marmosets (*Callithrix jacchus*),

high ranking individuals were found to have higher stress levels^8^ due to the demands of maintaining their high rank status^1,4,9^. Still, little is known about this relationship in more egalitarian social systems, although a recent study has shown that macaque species exhibiting a more egalitarian social model have generally higher stress levels compared to more despotic macaque species, which may be due to more uncertainty regarding social relations^10^. In humans, it has been shown that not only social but also socio-economic status (SES) is strongly linked to many health indices, including stress levels^11^. For instance, being raised in disadvantaged SES conditions is related to shorter life expectancy and poorer physical health, such as reduced inflammatory response due to acute psychological stress in adulthood^12–15^. Studies suggest that SES, like rank, is directly associated with social support, such as emotional and instrumental (e.g., material) support^16^ which, in turn, are associated with health outcomes^17^.

So far, most studies looking at the relationship between social status and health are derived from large-scale, hierarchical societies^4,17^. However, during the vast majority of humans’ evolutionary past people lived in small-scale egalitarian societies similar to those observed among modern day hunter-gathers, such as the Ache in Paraguay^18,19^ and the Batek in Malaysia^20,21^. As opposed to large-scale, hierarchical societies, immediate-return (i.e., an economic system where “people obtain a direct and immediate return from their labour…” such as “…hunting or gathering and eat the food obtained the same day or casually over the days that follow”^22^) hunter-gatherers have very little personal possessions and do not store any food surplus^22,23^, and it has been argued that in such egalitarian societies social status is more related to social prestige, such as popularity and foraging reputation, rather than social influence induced by material wealth^24^.

The link between popularity (i.e., being perceived as a friend by the others) and social status has been widely explored in large-scale, hierarchical societies^25^. It has been shown, for instance, that popularity is not only an important domain of social status but also a critical component of social integration (i.e., the attachment an individual sustains with the larger society^26^)^25^. It has been argued that in large-scale, hierarchical societies individuals who are better integrated in friendship networks have better access to social support resulting in improved indices of wellbeing^27^. Indeed, there is a growing body of evidence showing that popularity is associated with many health outcomes^27–29^. For instance, less popular individuals experience higher levels of anxiety related to social rejection^30^, have increased odds of suffering from depression^31^, suffer from higher infection risk^32^ and exhibit higher levels of physiological stress, such as blood fibrinogen^27^ compared to more popular individuals. Interestingly, in one of the handful of studies investigating perceived friendship relationships among Hadza woman, popularity was found to be unrelated to physiological stress^24^.

In contrast to popularity, the importance of hunting reputation has been widely covered in the hunter-gatherer literature, although it has been discussed mainly in terms of reproductive success^20,33,34^ rather than wellbeing. Moreover, the few studies looking at the relationship between wellbeing and hunting reputation among men living in traditional hunter-gatherer societies have been mostly based on basic anthropometric measures, such as BMI, upper body strength and voice pitch^35–37^ rather than long-term physiological indices of health. Little is known regarding the extent to which hunting reputation is related to long term indices of wellbeing, such as stress levels, among men in small scale traditional societies.

Here, we aimed to determine the extent to which social status (popularity and reputation) among Hadza men, hunter-gatherers living in northern Tanzania, is associated with hair cortisol concentration (HCC), a measure of wellbeing. Although HCC is not regarded as a synonym of stress per se^38^, it has been suggested to be a reliable indicator of ongoing chronic stress^39,40^ and has been successfully used as an indicator of wellbeing among both human and non-human primates^10,24,41^. We have recently shown that, among the Hadza, HCC is associated with proximity to other camp members and self-perceived friends^42^. Moreover, HCC levels have been found to be associated with self-perceived^43,44^ and physiological indices of wellbeing^45^ especially among those raised in high-risk conditions^46^. For example, high HCC values were associated with poorer self-perceived health and mental health in people living in industrialised contexts^40,47^ (but see^48^) as well as acute myocardial infarction (AMI^45^). Higher levels of HCC have been also linked to poor social engagement among people with dementia^49^.

The Hadza are perfect study participants for investigating the interplay between social status and health because they live in a mostly egalitarian society and thus show very little variation between individuals in terms of material wealth and a lack of formalized leadership^50^. This allowed us to assess the effects of popularity and reputation on physiological stress levels in the absence of wealth disparity (and other potentially confounding variables linked to this).

In a previous study we have shown that among Hadza women HCC was not associated with either popularity or foraging reputation^24^. However, considering sex differences regarding foraging activities among the Hadza, these two domains of social status might have different social significance for men and women, especially in terms of foraging reputation. Hadza women are predominantly involved in gathering plants (e.g., digging tubers and harvesting berries) while men mainly hunt and collect honey^51,52^. Thus, given that the Hadza value higher meat and honey over plants regardless of sex^53^, social (and health) benefits accrued by hunting reputation might be higher in men compared to women. This could lead to either a positive or a negative association between hunting reputation and HCC.

Although we did not make specific predictions regarding the direction of the effects, we explored different scenarios. A positive relationship would suggest that better hunters are either under more social pressure (e.g., expectations regarding hunting success/food provision) or that the physiologically demanding hunting activity is reflected in elevated stress levels. On the other hand, a negative association between hunting reputation and HCC would potentially suggest that either prestige accrued by being perceived as a good hunter provides social benefits, such as better opportunities for social partners and/or, given that Hadza hunters consume the majority of the most valued food while out-of-camp hunting^51^, being a good hunter might provide nutritional benefits as well. Regarding the effects of popularity on HCC in Hadza men, one would predict a negative relationship, given that being more popular might provide a better access to social buffering^29^. However, this relationship may not hold true for the Hadza, as it has rarely been tested in small-scale egalitarian societies^24^. Moreover, a recent study suggests that living without a clear dominance hierarchy may in itself be stressful^10^.

## Results

**Figure 1:**
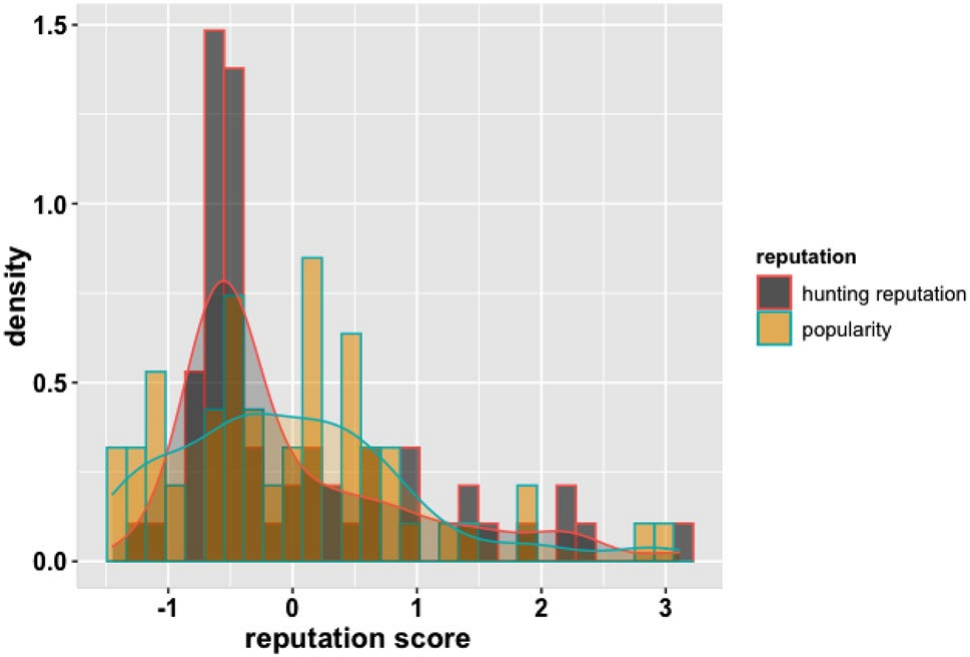
Histogram and cumulative density distribution of reputations: foraging and popularity. X axis is the within-camp z score for each reputation metric for each man.

**Figure 2:**
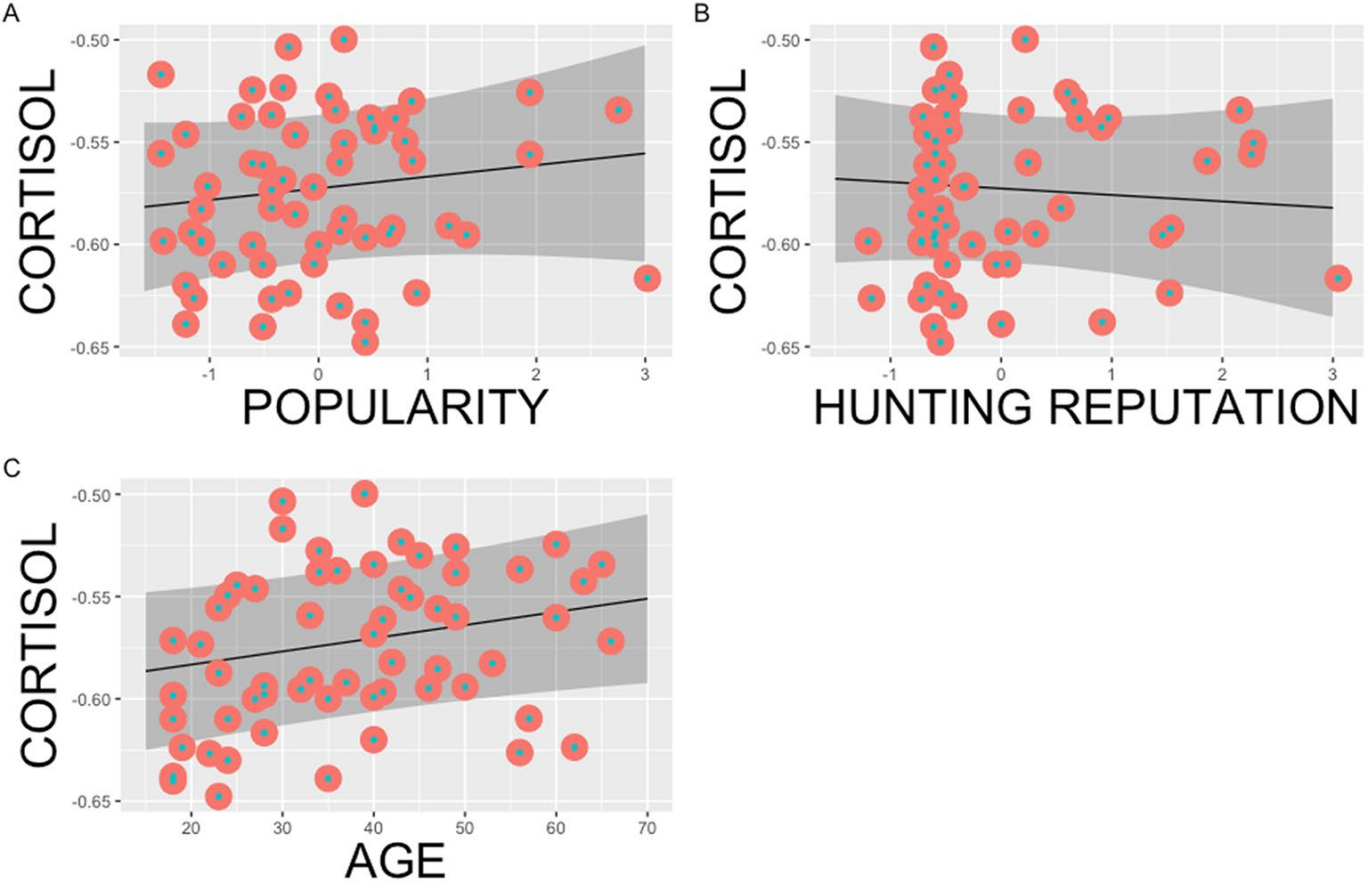
Popularity, hunting reputation and age associations with cortisol. The relationship between power transformed picograms of hair cortisol concentrations and within-camp z score for each reputation metric for each man (**A**) is popularity, (**B**) hunting reputation and (**C**) age. Shaded area represents predicted 95% confidence intervals derived from the LMM model.

Our cortisol sample consisted of 60 men, with mean hair cortisol concentration of 96.82 pg/mg (median = 80.73, range 32.26–257.14). A Wilcoxon Rank Sum Test found significant differences in HCC between the two data collection field seasons (W = 682; p < 0.001, n = 2). Both popularity and foraging reputation scores exhibited a positive skew, especially in bigger camps (Table 1, Supplementary Material) showing that a small number of individuals received the majority of nominations for both measures in each camp (Fig. 1, Figs. 1a–h, 2a–h, Supplementary Material).

**Table 1:** Kendall correlation results between variables included in the model. All values, except age, are z-scored within their respected camps.

	Hunting reputation	Popularity
Age	Tau = 0.174, p = 0.053	Tau = 0.127, p = 0.160
Hunting reputation		Tau = 0.51, p < 0.001

Although some model variables were significantly correlated with each other (Table 1), because Kendall Tau and all VIF values were generally low (age VIF = 1.06, hunting reputation VIF = 2.39, popularity VIF = 2.36), all variables were included in the full model.

Neither hunting reputation nor popularity were associated significantly with hair cortisol concentrations (although age was marginally significant) (Table 2; Fig. 2) also when popularity and hunting reputation were included separately for the LMM analyses (Tables 2S and 3S, Supplementary Material).

**Table 2:** LMM results explaining cortisol concentration variance among the Hadza man.

	*B* ± SE	t value	Pr( > |t|)	AIC	R^2^
Intercept	− 0.5960 ± 0.219	− 27.24	0.001	− 188.4	Marginal: 0.055
Age	0.0006 ± 0.004	2.016	0.049		Conditional: 0.43
Popularity	0.0568 ± 0.092	0.866	0.390		
Hunting reputation	− 0.0031 ± 0.094	− 0.469	0.641

## Discussion

The results of our study show that neither hunting reputation nor popularity is related to stress among Hadza men. As such, the results of our study suggest that, at least in some small societies presenting an egalitarian social model, such as the Hadza, social status does not have the effects on stress that might be expected given work on large-scale hierarchical societies. Our data are consistent with our previous study that found that foraging reputation and popularity is not related to physiological stress levels among Hadza women^24^, although lack of association between hunting reputation and HCC is rather surprising given the social significance of the former in small-scale egalitarian hunter-gatherer societies^33,54,55^.

Although the extent to which hunting reputation reflects the actual hunting skills of Hadza men has been debated^56^, good hunters tend to be perceived as preferred husbands and campmates^23,57^ and, thus, hunting reputation is socially significant^58^. Moreover, hunting skills might not only accrue social benefits, such as social prestige, but also have nutritional value and therefore, directly contribute to their health. For instance, it has been shown that Hadza men consume most of the food while foraging and only fraction is brought to the camp^51^. Moreover, in non-industrialized societies hunting reputation has been found to be associated with upper body strength, which itself is linked to reproductive success^36^, while friendship relationships have been argued to serve as a sort of social support insurance at times when individuals need it^59^. It has been argued that friendship relationships play an important role by providing, for instance, security against uncertain ecological conditions^60–62^ as food resources, such as big game, are rarely obtained predictably^23,51^. Therefore, the results of our study showing that social status is not related to stress might be rather surprising.

One of the reasons for the absence of a relationship between hunting reputation and physiological stress levels in our study could be that Hadza men do not get stressed to the extent that would be reliably reflected in physiological chronic stress indices, such as HCC. Indeed, the Hadza often describe their life as ‘relaxed’ [e.g., when asked whether they experience any worries or anxieties, they usually respond in Swahili ‘hamna shida’ (no worries/there are no problems)] while weak constraints on their social mobility coupled with an egalitarian social model and widely known food sharing might provide an effective buffering against both food insecurity and potential social tensions between camp members^23,50^. Indeed, malnutrition is nearly absent among the Hadza while killings of Hadza by Hadza, although reported, are rare^63^. Moreover, the Hadza are relatively healthy, with absence of nutritional marasmus, kwashiorkor, scurvy rickets, and vitamin B deficiency syndrome^63^. Nevertheless, chronic conjunctivitis, malaria, oral diseases and, most of all, infectious diseases caused by infected wounds (e.g., resulting from scorpion bites) are common among the Hadza^23,63,64^. Infected wounds, for instance, often require medical intervention and pose a serious health risk when untreated (personal observation) and streptococcal infections have been reported to be a significant cause of death among the Hadza^65^. In addition, injuries resulting from tree falls are not infrequent^23,63^. Apart from health issues, Hadza often express their concern regarding water availability. For example, while collecting data for this study the lack of easy access to water was a major concern of the Hadza in three out of eight camps. In addition, during the interviews Hadza men frequently expressed their concerns regarding the diminishing number of wild animals in hunting areas as well as an increasing influx of pastoralists Datoga people into their land that has occurred in recent years (personal observation).

Another reason for the apparent lack of association between social status and stress levels among Hadza men could be that hair cortisol levels, or more generally glucocorticoids levels, may not be synonymous with stress^38^. Indeed, it has been argued that cortisol and glucocorticoids have several different roles in signalling beyond stress reactions^40^. However, although the relationship between cortisol and wellbeing might be less straightforward than previously believed^38^, most recent human studies confirm that HCC is a reliable indicator of long-term wellbeing^66,67^. In addition, Hadza men keep their hair short and thus HCC levels will present only the last few weeks of stress levels. Although we collected our data on popularity and reputation within the same time frame, if may be that any differences in HCC were too subtle and/or not present during the time of data collection.

Yet another reason could be that individuals’ hunting reputations cannot be established reliably based on in-camp interviews due to the flexible residence pattern of the Hadza. Over the course of a year, Hadza change camps on average six times^23^. Therefore, the inter-camp relationships might also play an important role in terms of social support^68^. Nevertheless, the inter-camp mobility observed among the Hadza also means that individuals are very well aware of the social status/reputation of Hadza members from neighbouring camps^50^. For example, they often eagerly express their opinions regarding the foraging skills of Hadza living in other camps (personal observation by PF). Indeed, it has been recently shown that preferences for individuals perceived as generous, such as successful hunters, are a factor behind inter-camp residency dynamics^57^, suggesting that individuals’ reputations are not solely limited to in-camp relationships but rather also have an inter-camp dimension (also see^69^). Therefore, Hadza moving to a new camp are most certainly perfectly aware of the social reputation of the camp residents^57^. It also could be argued that the measure of the two domains of social status provide only a partial picture of social status given that nominations were derived from within-camp nominations only. However, we have previously shown that the Hadza prefer to interact with their nominated friends (rather than non-friends) while in-camp derived hunting status is related to the position an individual maintains in proximity networks even when friendship/hunting reputation nominations are restricted to people living in the same camps^70^, further suggesting that the in-camp friendship nominations is a valid tool in estimating popularity status among the Hadza.

Still, considering the constant movement of the Hadza members between different camps, social reputation of the Hadza men may vary depending on the camp composition they are currently residing in, which is in a constant flux^23^. Thus, given that the hair derived cortisol covers the last 1–2 months and that the composition of the study camps might have changed during this period before the study commenced, the influence of the collected social status on stress levels might not be accurately reflected in our study (especially given the small sample size of our study). This might be especially true regarding (the within-camp) popularity as hunting reputation has inter-camp recognition^57^. Nevertheless, it has been shown that the Hadza do not choose their camps at random and their camp choices are influenced by social preferences based on friendship and hunting reputation of their campmates^57^ suggesting that they might follow their friends/kin while changing a camp. Equally, if the Hadza men had been residing in a camp without social support we would expect this to be reflected in HCC. During the time of data collection very little camp movement occurred, so that HCC are mostly based on the current residency choices.

Lastly, our social status data collection method may have affected the results. In our study we concentrated on the ‘three best’ hunters/friends, instead of grading all camp members. This may have skewed the data with a small proportion of men receiving most of the ‘best hunter’ nominations while a large proportion (~ 30%) did not get any nominations. However, even in an egalitarian society a skewed distribution of nomination scores is expected, especially for hunting reputation (as there will only be a few best hunters). Furthermore, this method has been used in other studies (e.g.,^29,70,71^) and should have enabled us to detect any main effects if present. Moreover, when it comes to social status, an egalitarian society is characterised by the absence of a linear grading system^23,50,63^, although status differential can be found using the method we used^70^. Alternatively, the apparent lack of a relationship between HCC and social status among the Hadza men recorded in this study is a consequence of their egalitarian system, in which there is a negligible variation of material wealth among campmates and food is generally widely shared^23,50^. Thus, although individual hunting skills in egalitarian small-scale societies, such as the Hadza, are recognised^20,72^, they do not translate into differences in resources. Individual autonomy is highly respected across all ages among the Hadza whereas competition and coercion are consistently supressed^50^, resulting in a strong social convention that discourages successful hunters from exploiting their hunting prowess^23^. Similar forms of levelling mechanisms have been also recorded in other small-scale egalitarian societies, such as the Batek of Malaysia^20^.

It has been suggested that the levelling mechanisms observed in highly egalitarian small-scale societies can moot some of the effects that hunting reputation can have not only on health but also reproductive outcomes^20^. Although a relationship between hunting success or reputation and reproductive success has been documented in many traditional small-scale societies^73^, this association is less clear in more egalitarian societies, such as the Batek and Hadza^20,23^. For example, among the Batek both sharing proclivity and foraging return rate (or cooperative foraging) have been found to be poor predictors of lifetime reproductive success^20^. Therefore, given the level of variation in terms of subsistence among non-industrial societies^20^, the association between hunting reputation and stress would be more pronounced in traditional but less egalitarian non-industrial societies where the relationship between social status and social influence is less obscure compared to the Hadza (although in the latter men with better hunting reputation had younger wives^72^). Levelling mechanisms, which tend to underplay the differences in ability among individuals, may be especially apparent among subsistence hunter-gatherers, such as the Batek, Hadza, ByYaka of Central Africa and Chewong of Malaysia^74^, where food uncertainty and food sharing are widely observed. It has been argued that, among highly egalitarian hunter-gatherers, risk pooling strategies, such as a collective contributions in obtaining unpredictable food resources^62,75^, are promoted by such a variance in foraging skills among camp mates^24^ which, in turn, contributes to food sharing and, therefore, enhances social cohesion^75^.

Although our data further strengthen the argument that health benefits related to social status might not necessarily be universal across human all societies^24^, caution should be taken regarding the extent to which the results of this study can be extrapolated to contemporary and early pre-historic hunter-gatherers. This is because the egalitarian social system exhibited by the Hadza, with relatively peaceful lifestyle and widely shared food, might be representative of neither extant nor early pre-historic small-scale hunter-gatherers. For example, as opposed to the Hadza, among the !Kung of Botswana, a contemporary small-scale hunter-gatherer society exhibiting a relatively egalitarian social model, the homicide rate between 1920 and 1955 was high, about 4 times higher compared to the current U.S. rate^76^, while food insecurity is a major source of distress among indigenous Inuit of Northern Canada^77^ who have been reported to solve their problems with violence^78^. It is, therefore, possible that the relationship between social status, as measured by popularity and hunting reputation, and stress might differ in these two hunter-gatherer groups compared to what our data show. Furthermore, the extent to which a highly egalitarian, mobile and relatively non-violent (e.g., with a lack of warfare) social model of the Hadza is representative of pre-industrial hunter-gatherers is debatable^79,80^. Therefore, caution should be taken regarding the degree to which extant hunter-gather ethnic groups, such as the Hadza, can be used in order to infer things about the human evolutionary past^42,81^.

Overall, in this study we show that, among Hadza men, social status does not correlate with lower levels of cortisol. We have previously shown that, among Hadza women, neither popularity nor foraging reputation is related to stress levels^24^ and the current study only strengthens our previous finding showing that the relationship between status and markers of health such as physiological stress, might not be universal across societies. As such, the results of our study might have important implications for research looking at the relationship between social status and health in humans.

## Methods

### Study population

The Hadza live in very fluid, mobile camps of roughly 30 adults, and camp composition changes substantially over the course of a year^50,63^. It is estimated that there are about 1000 Hadza left in Tanzania but only about 300 of them continue to exhibit a hunter-gatherer lifestyle^23^. There is a clear sex division with respect to daily activities of the Hadza, with men usually dedicated to hunting and honey collection while women are usually involved in childcare and the gathering of fruits and other types of plants^23,51^.

### Data collection

Data were collected in 2016 and 2017 for roughly four months each. Data collection was conducted in camps that were actively foraging for most of their calories. Nevertheless, since CB’s first visit in 2007, limited trade with neighbouring food-producing ethnic groups, mainly Datoga pastoralists, has been occasionally observed in most camps. Because the Hadza generally do not know their precise birth year, their age was established through long term records established by Nicholas Blurton Jones^63^ and maintained by Frank W. Marlowe^23^ and JCB. Data collection lasted a few weeks in each camp, with hair sampling conducted on the final day of data collection so that hair sampled grew during the time when camp members were co-resident with the other members they were nominating.

Hair samples and reputational data on popularity status and foraging reputation were collected from eight camps of different sizes (ranging from 6 to 38 adults, mean = 19; number of men in a camp ranged from 2 to 17, mean = 8.12). All adult women (n = 83) and men (n = 64) present in every camp participated in the study. We collected reputational data from 100% of adults per camp and hair samples from 62 men. Out of the 147 study participants, 2 men and 3 women were sampled twice as they happened to be in two different study camps when the study was conducted^24^. The hair samples from the first camp only were used for the women sampled twice, but nominations provided in both camps were used (more detail below). Supposing that only approximately 250 Hadza are full-time foragers, and roughly half of those are men, our data set constitutes a fairly representative sample of one of the last remaining egalitarian hunter-gatherer populations^24^.

### Reputation measures

To establish Hadza men’s reputations for competency in a socially valued skill, we asked 147 adult camp members (both men and women resident in camp), in private, one at a time, who they considered the three best hunters in camp. Interviews with the Hadza were conducted by the PI in Swahili, in which the majority of the Hadza are fluent, although some older Hadza required the assistance of an interpreter (i.e., a Hadza research assistant fluent in the Hadza language) during the interviews as their command of Swahili was basic. The interviews were conducted during the last day of the study in a camp in a secluded area just outside of the camp so that interviews were out of earshot of other people in the camp. Because only men are involved in hunting activities, both women and men nominated only men as the top three best hunters. We collected reputation measures on 64 men, with a mean age of 38.31 years (median = 38, range 18–66).

During the same interviews described above, all respondents (n = 147 adults, 83 women, and 64 men) were also asked who their three best friends in that camp were. Even though men were free to nominate friends regardless their sex, out of 64 men all but two named women as their three best friends in camp, and only three women named a man as a friend.

While scoring reputation status for each study participant, we assigned 3 points to the top score (such as 1st best friend/1st best hunter), 2 points to the second and 1 point to the third best nominee. Because we collected reputational data from eight camps of different sizes with different numbers of potential nominators, we standardized these raw values by deducting a mean camp value from each individual score in a camp and then dividing it by the standard deviation of the camp—which resulted in a within-camp z score for each reputation metric for each woman^82^. In addition, we calculated the skewness of the nominations scores using the e1071^83^ package for R^84^, where positive and negative values indicate positive and negative skew respectively.

### Cortisol analysis

We are mainly interested in chronic longer-term levels of stress rather than the acute stress response because we are interested in the mediation of chronic stress. Hair cortisol has the advantage of allowing back-tracking average levels of cortisol over a longer time-frame than other methods (such as salivary cortisol); depending on hair length and growth rate, cortisol can be reflecting stress levels experienced over the last few months^85^. Assuming an average hair growth rate of 1 cm/month^85^, the record of hair cortisol in our samples covered the last 1–2 months, which corresponds roughly to the time documented as the average duration of Hadza residence in a particular camp^23^.

In addition, there are well-documented associations between cortisol levels, insulin resistance, hypertension, immunosuppression, and reproductive impairments. As a result, higher cortisol levels are associated with serious health implications, including reduced life expectancy^86,87^.

Determining cortisol from hair also has the benefit of being a biological sampling procedure that causes minimal discomfort to the participants and does not require refrigeration after collection (which would be quite difficult to have in mobile, fluid bush camps). It is worth noting that many Hadza women keep their hair short, so that the time period of cortisol levels detected in hair is estimated to be approximately one month. The Hadza do not dye or bleach their hair. They occasionally use soap (generally hand soap) to wash their hair, so cortisol values were unlikely to be affected by frequent shampooing seen in other cultures^88^.

### Hair preparation and analysis

Hair cortisol was extracted according to Sauvé et al.^89^. Briefly, hair samples from the 1 cm closest to the scalp end were cut into small pieces using sterile small surgical scissors^90^, weighed (to around 10–15 mg) and placed into 1.5 ml reaction tubes^24^. Prior to extraction, hair samples were ground using the IKA ULTRA TURRAX Tube drive System (following^91^. For extraction, 1.5 mL of methanol was added, and the vial was sealed and incubated overnight for 18 h at room temperature while gently shaking. After incubation, samples were centrifuged, the methanol extract was transferred to a disposable glass vial and evaporated to dryness under nitrogen^24^. The samples were dissolved in 250 μL phosphate buffered saline (pH 8.0). Samples were vortexed for one minute, and then again for 30 s before the assay.

Cortisol levels were measured using the Salimetrics® Cortisol Enzyme-linked Immunoassay (ELISA) Kit (Salimetrics Europe, Suffolk, UK) as per manufacturer’s instructions. In principle, the assay measures competitive binding to a capture antibody between hair-extracted cortisol and cortisol conjugated to horseradish peroxidase, which converts 3,3',5,5'-tetramethylbenzidine (TMB) to 3,3',5,5'-tetramethylbenzidine diimine in a chromogenic reaction. After termination of the reaction by adding sulphuric acid, absorbance was measured at 450 nm and cortisol levels were calculated based on a standard curve. The intra- and inter-assay coefficient of variance was < 9% and < 10% respectively.

### Statistical analysis

Because HCC was highly skewed, we transformed these measurements^24^ using the Tukey Ladder of Power procedures, which applies a power transformation on a data set^92^ using the R companion package^93^. Following that, visual inspections of normality and homogeneity of error variances did not indicate a violation of model assumptions.

Data were analysed using linear mixed-effects model (LMM) fit by the restricted maximum likelihood estimation (REML) with cortisol levels as a dependent variable while hunting reputation and popularity status were used as predictors. Regarding the two men that participated in the study twice in two different camps, for the LMM analysis we used only data from the first camp they were sampled in order to avoid pseudo-replication (although their nominations were included in quantifying the popularity and hunting reputation of other participants, they were nominated in both camps in which they participated^24^). In addition, all men (n = 2) in one of the comps did not receive any friendship nominations and therefore were not included in the LMM analyses (as z-scoring their friendship nomination scores resulted in generating undefined (NA) values). We also included in the model age as a fixed independent variable and camp nested within a field season (as Wilcoxon Rank Sum Test showed that there were significant HCC differences depending on the field season (n = 2; e.g., 2016 and 2017 during which the hair samples were collected) as a random variable.

To minimize the problem of collinearity, we first ran Kendal Tau correlations on all variable combinations to avoid including highly correlated variables (Kendal tau > 0.8) in the model. We also calculated variance inflation factors (VIF) for all the variables, including only variables with VIF < 4. We calculated marginal (i.e., for fixed effects only) and conditional (i.e., for both fixed and random effects for camp) R^2^ for the LMM model using the ‘lmerTest’ package^94^ for R. LMM was performed using the ‘lme4’package^95^ for R^84^. Figure 2 was generated using the ‘ggpredict’ function available in the ‘ggeffects’ package^96^ for R. Because popularity and hunting reputation were correlated with each other (tau = 0.51, *p* < 0.001), we also calculated two separate LMM models: one with all variables retained from the original model but without popularity and the other with all variables retained from the original model but excluding hunting reputation (see Supplementary Material, Tables S2 and S3), following^24^.

In addition, we used a Wilcoxon Rank Sum Test in order to determine whether HCC values differed between the two field seasons during which the hair samples were collected.

### Ethical statement

Informed consent was obtained from all study participants. This research was approved by University of Roehampton ethics committee (LSC 15/118), as well as by Tanzanian authorities COSTECH and NIMR. The study was performed in accordance with the Declaration of Helsinki as well as other relevant guidelines and regulations.

## Supplementary Information

Supplementary Information.

## Data Availability

The datasets generated during the current study are not publicly available in order to protect the anonymity of health data from the Hadza but a simulated dataset generated by the R package synthpop^97^ will be made available in the Figshare data depository.
