# Supplementary material for: Status does not predict stress among Hadza hunter-gatherer men
Source: Sci Rep. 2023 Jan 24;13:1327. doi: 10.1038/s41598-023-28119-9 (PMC9873806; doi:10.1038/s41598-023-28119-9)
Supplement: Supplementary file 1 — Supplementary Information. [file 41598_2023_28119_MOESM1_ESM.pdf]

Supplementary Material

Table 1S Skewness values of Popularity and Hunting reputation scores

	Camp A	Camp B	Camp C	Camp D	Camp E	Camp F	Camp G	Camp H
Popularity	0.59	1.33	-0.03	0.49	0.68	0.32	-0.15	0
Hunting reputation	0.66	2.56	0.37	1.75	0.76	0.72	0.72	0.22

Table 2S LMM results with popularity excluded.

	B ± SE	t value	Pr(>|t|)	AIC	R^2
Intercept	-0.597±0.022	-27.461	0.001	-197.91	Marginal: 0.05
Age	0.001 ± 0.0003	2.057	0.044		Conditional: 0.42
Hunting reputation	0.001 ± 0.004	0.268	0.789		

Table 3S LMM results with hunting reputation excluded.

	B ± SE	t value	Pr(>|t|)	AIC	R^2
Intercept	-0.595 ± 0.22	-27.39	0.001	-198.4	Marginal: 0.05
Age	0.0006 ± 0.0003	1.979	0.053		Conditional: 0.43
Popularity	0.003± 0.004	0.784	0.436		

In Figures 1a-h, we show the number of individuals in each camp to receive nominations for being the best hunter. Higher nomination scores indicate the number of nominations for being the best hunter. Number of participants refers to how many people in camp received each nomination score (best hunter = 3 points, 2nd best hunter=2 points, third best hunter = 1 point, not nominated = 0 points).

In Camp 1, for example, four people were never nominated by a single person as being in the top 3 hunters in camp. One person was named so often they had a score of 50.

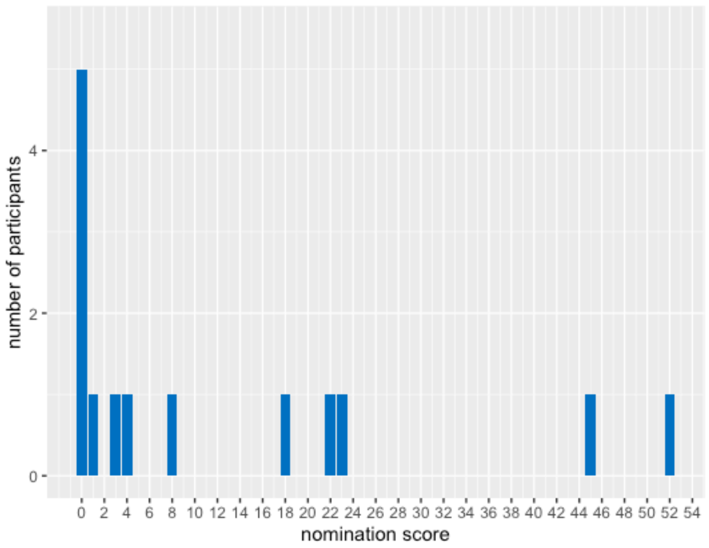

Figure 1a. Camp 1 Hunting nomination breakdown (camp n hunters = 14, n nominators = 33).

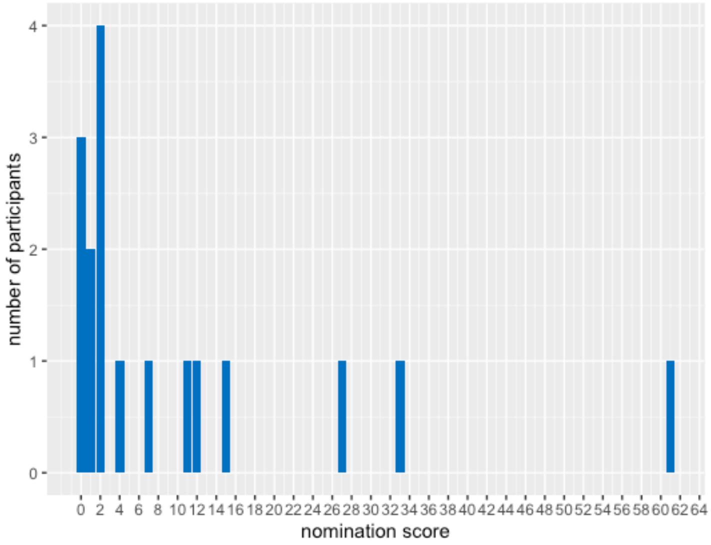

Figure 1b. Camp 2 Hunting nomination breakdown (camp n hunters = 17, n nominators =38)

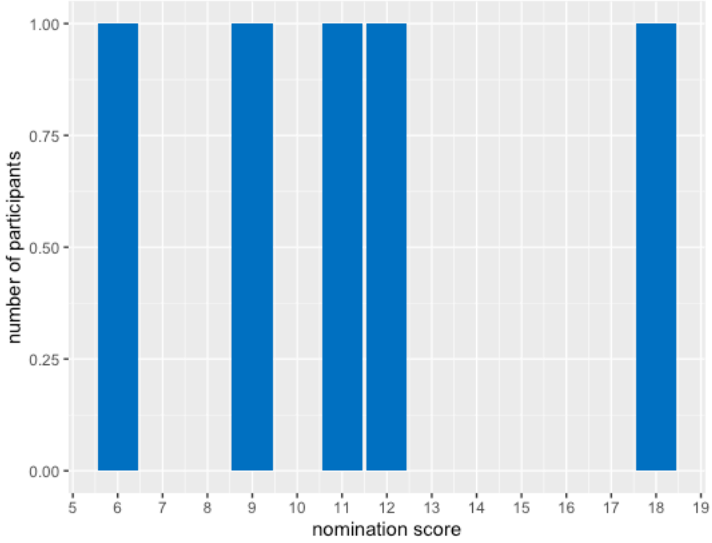

Figure 1c. Camp 3 Hunting nomination breakdown (camp n hunters = 5, n nominators = 12)

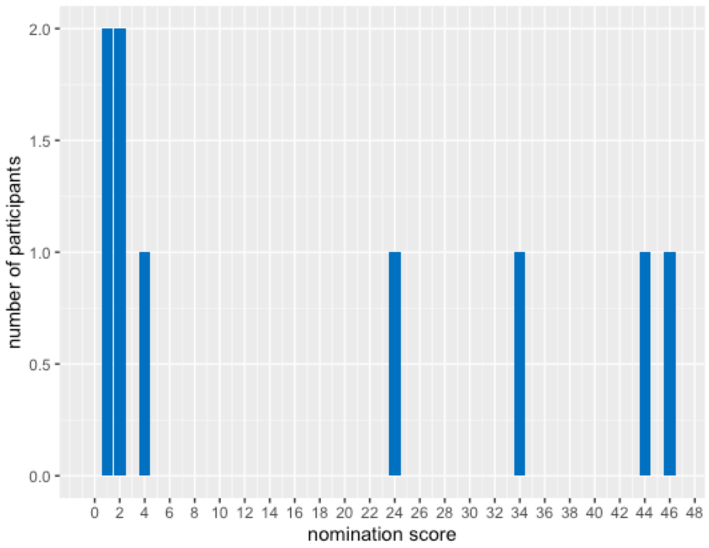

Figure 1d. Camp 4 Hunting nomination breakdown (camp n hunters = 17, n nominators =34)

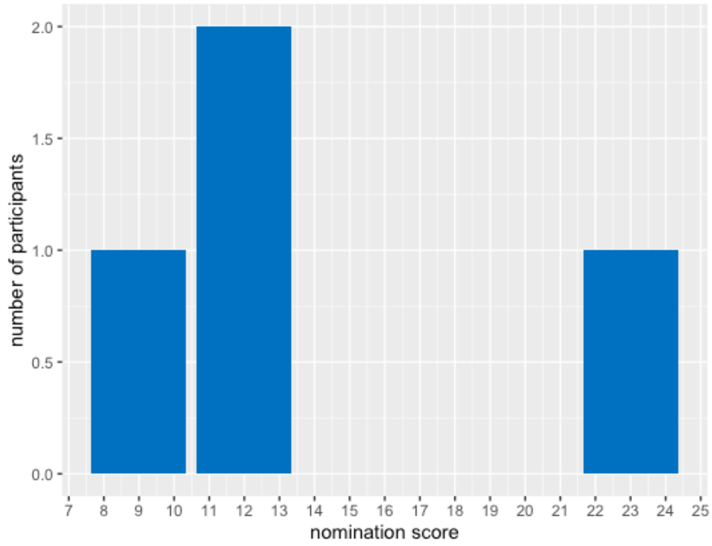

Figure 1e. Camp 5 Hunting nomination breakdown (camp n hunters = 4, n nominators = 10)

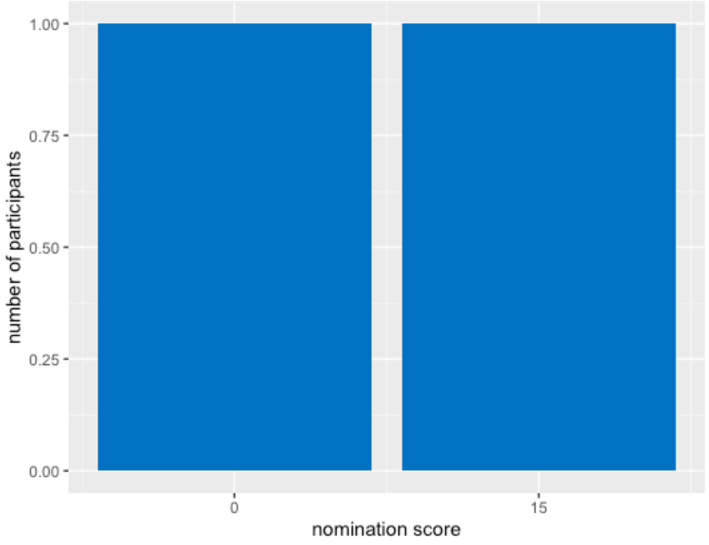

Figure 1f. Camp 6 Hunting nomination breakdown (camp n hunters = 2, n nominators = 6)

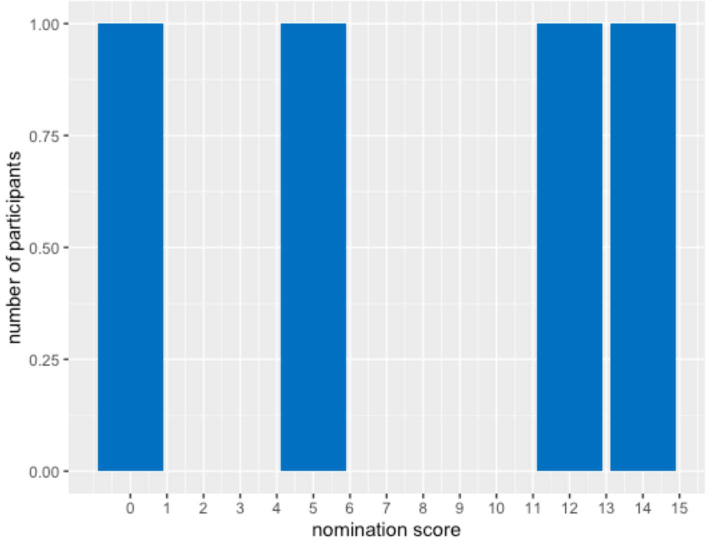

Figure 1g. Camp 7 Hunting nomination breakdown (camp n hunters = 4, n nominators = 7)

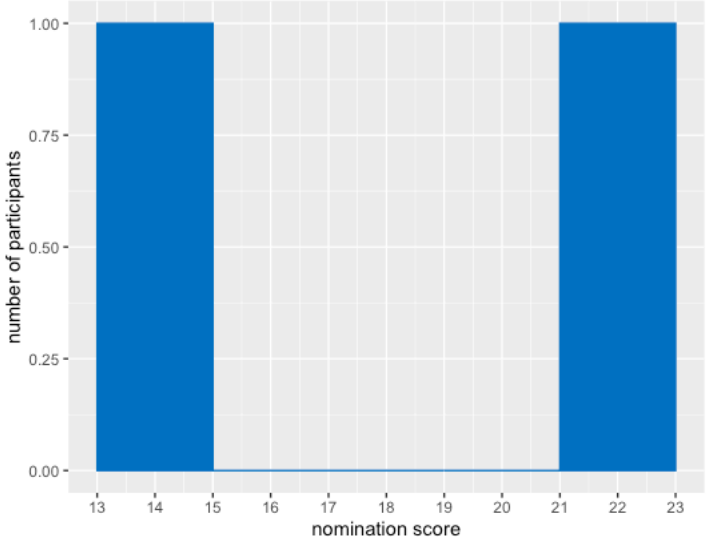

Figure 1h. Camp 8 Hunting nomination breakdown (camp n hunters = 2, n nominators = 12)

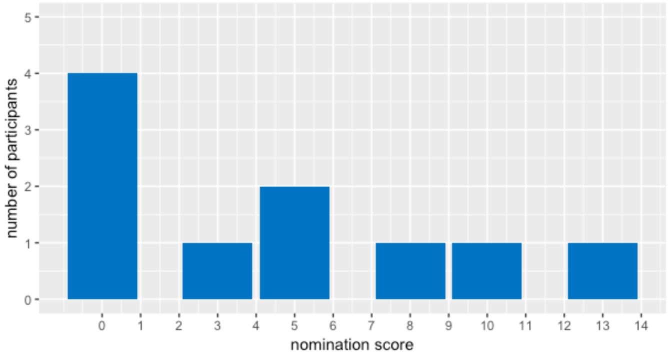

In Figures 2 a-h we show the number of individuals in camp to receive nominations for being the best friends of others. Higher nomination scores indicate the number of nominations for being the among someone's named three best friends. Number of participants refers to

how many people in camp received each nomination score (best friend = 3 points, 2nd best friend =2 points, third best friend = 1 point, not nominated = 0 points).
In Camp 1, for example, four people were never named among anyone's three best friends, and one person received a score of 15.

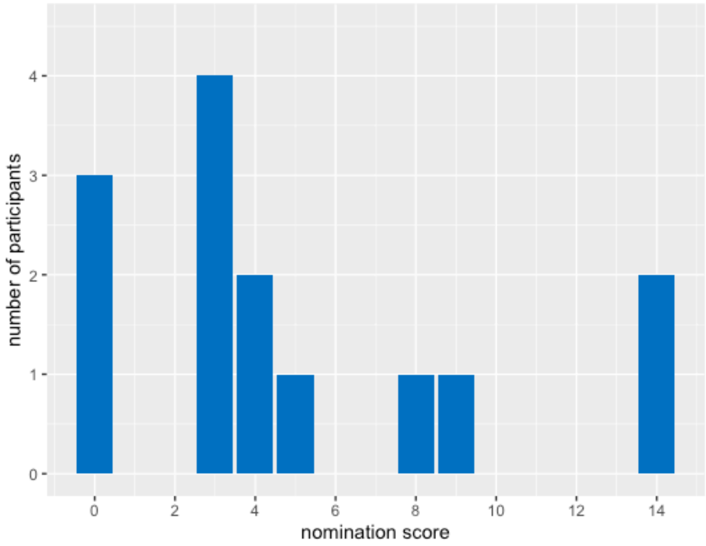

Figure 2a. Camp 1 Friendship nomination breakdown (camp n potential friend nominees = 14, n nominators = 33)

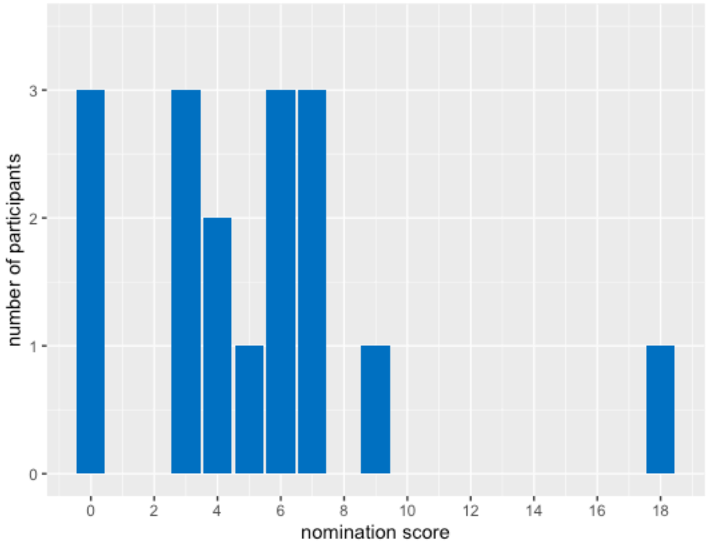

Figure 2b. Camp 2 Friendship nomination breakdown (camp n potential friend nominees = 17, n nominators = 38)

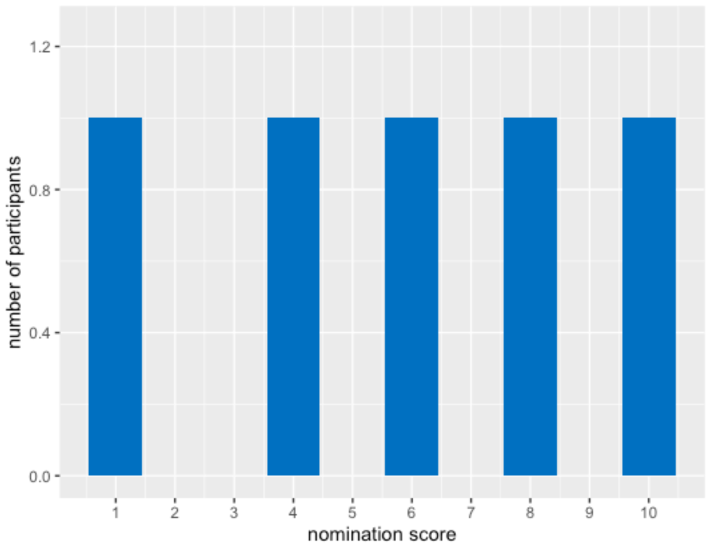

Figure 2c. Camp 3 Friendship nomination breakdown (camp n potential friend nominees = 5, n nominators = 12)

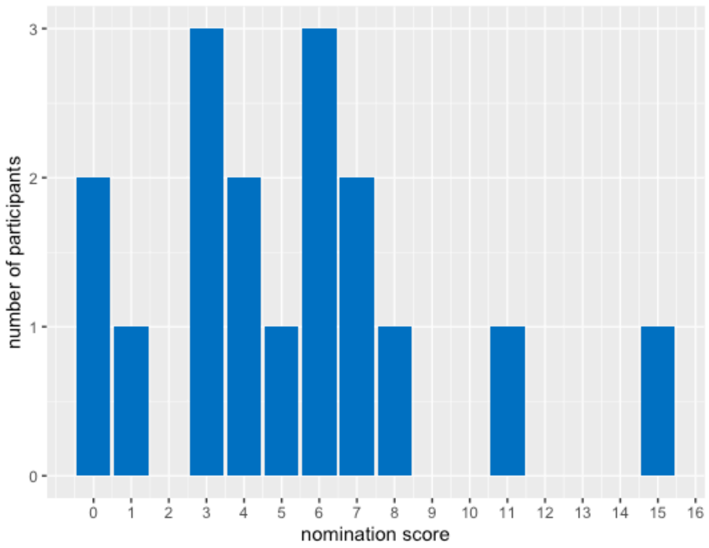

Figure 2d. Camp 4 Friendship nomination breakdown (camp n potential friend nominees = 17, n nominators = 34)

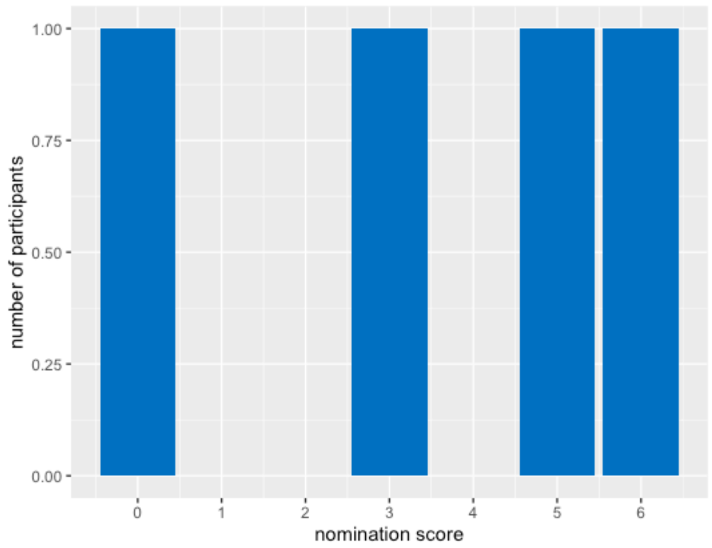

Figure 2e. Camp 5 Friendship nomination breakdown (camp n potential friend nominees =4, n nominators = 10)

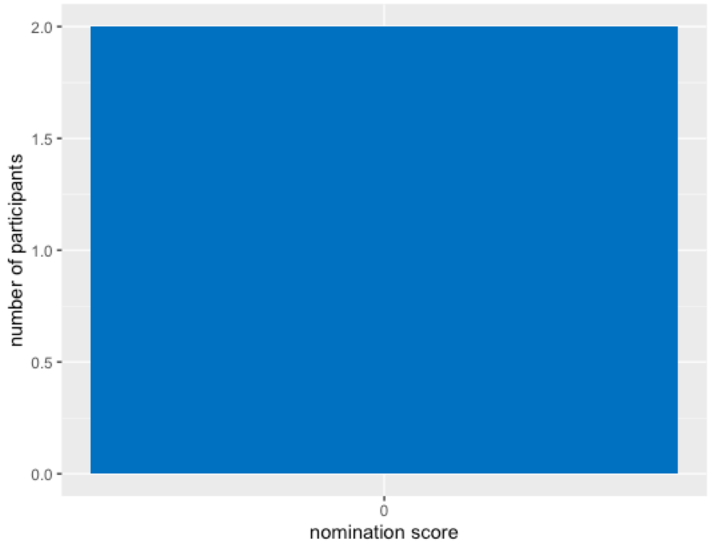

Figure 2f. Camp 6 Friendship nomination breakdown (camp n potential friend nominees = 2, n nominators = 6)

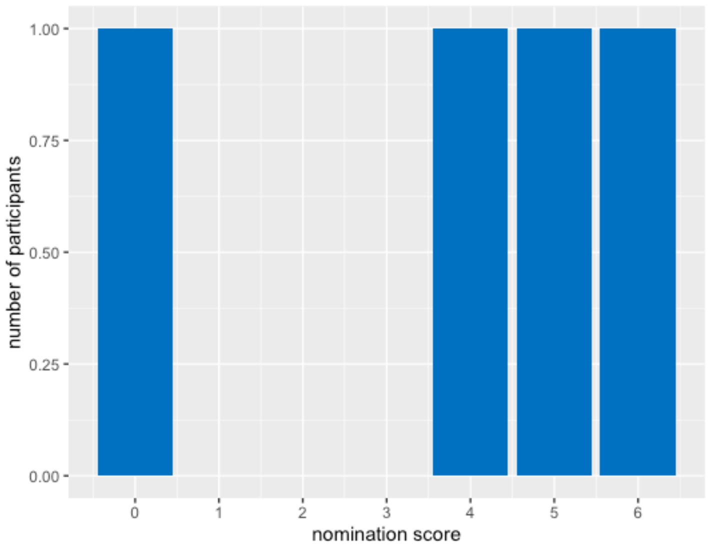

Figure 2g. Camp 7 Friendship nomination breakdown (camp n potential friend nominees = 2, n nominators = 7)

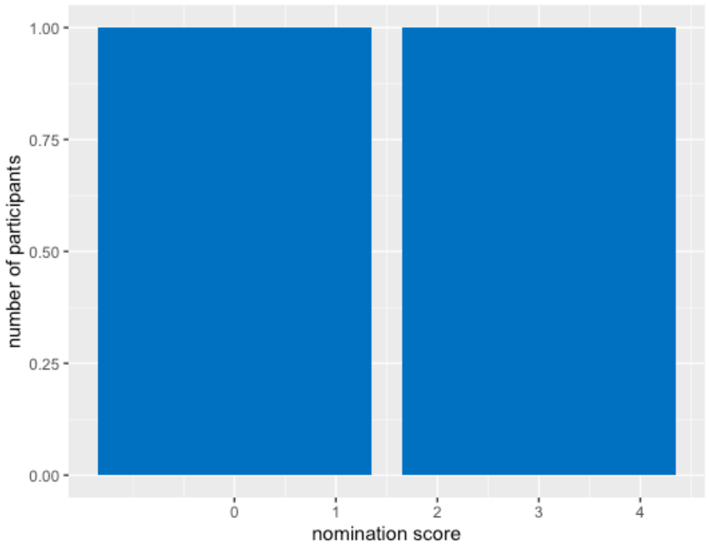

Figure 2h. Camp 8 Friendship nomination breakdown (camp n potential friend nominees = 2, n nominators = 12)
